# The local concentration of Ca^2+^ correlates with BMP7 expression and osseointegration in patients with total hip arthroplasty

**DOI:** 10.1186/s13018-020-02090-x

**Published:** 2020-11-30

**Authors:** Xiaodong Fu, Weili Wang, Xiaomiao Li, Yingjian Gao, Hao Li, Yi Shen

**Affiliations:** Department of Orthopedics, School of Medicine, Ren Ji Hospital, Shanghai Jiao Tong University, Shanghai, China

**Keywords:** Uncemented THA, Ca^2+^, BMP7, Stro-1^+^ cells, Osseointegration

## Abstract

**Background:**

A successful osseointegration of total hip arthroplasty (THA) relies on the interplay of implant surface and bone marrow microenvironment. This study was undertaken to investigate the impact of perioperative biochemical molecules (Ca^2+^, Mg^2+^, Zn^2+^, VD, PTH) on the bone marrow osteogenetic factors (BMP2, BMP7, Stro-1^+^ cells) in the metaphyseal region of the femoral head, and further on the bone mineral density (BMD) of Gruen R3.

**Methods:**

Bone marrow aspirates were obtained from the discarded metaphysis region of the femoral head in 51 patients with THA. Flow cytometry was used to measure the Stro-1^+^ expressing cells. ELISA was used to measure the concentrations of bone morphologic proteins (BMP2 and BMP7) and the content of TRACP5b in serum. TRAP staining was used to detect the osteoclast activity in the hip joint. The perioperative concentrations of the biochemical molecules above were measured by radioimmunoassay. The BMD of Gruen zone R3 was examined at 6 months after THA, using dual-energy X-ray absorptiometry (DEXA).

**Results:**

Our data demonstrated that the concentration of Ca^2+^ was positively correlated with BMP7 expression, and with the postoperative BMD of Gruen zone R3. However, the concentration of Mg^2+^ had little impact on the R3 BMD, although it was negatively correlated with the expression of BMP7. Osteoclast activity in hip joint tissue of patients with femoral neck fractures was increased. Compared with the patients before THA, the levels of TRACP5b in serum of patients after THA were decreased. The data also suggested that the other biochemical molecules, such as Zn^2+^, VD, and PTH, were not significantly correlated with any bone marrow osteogenetic factors (BMP2, BMP7, Stro-1^+^ cells). The postoperative R3 BMD of patients of different gender and age had no significant difference.

**Conclusions:**

These results indicate the local concentration of Ca^2+^ may be an indicator for the prognosis of THA patients.

## Background

Total hip arthroplasty (THA) is one of the most cost-effective and consistently successful surgeries performed in orthopedics. THA provides reliable outcomes for patients’ suffering from end-stage degenerative hip osteoarthritis, specifically pain relief, functional restoration, and overall improved quality of life [1]. THA patients must undergo initial stabilization to obtain long-term durability. Implant stability and long-term survival of THA require efficient osseointegration, which is a process requiring the recruitment of bone marrow mesenchymal stromal cells (BMSC) to the prosthetic surface. The commitment of BMSC cells to an osteoblastic differentiation pathway is apparently under the control of both systemic and local growth factors, such as bone morphogenic proteins (BMPs) [2, 3]. A favorable bone marrow microenvironment should have a sufficient amount of osteoprogenitor cells being able to differentiate to functional osteoblasts in response to growth factors. The differences in patient bone marrow microenvironments and the association of their bone marrow osseointegration potentials with post-THA outcomes have been long overlooked [4, 5].

Biochemical molecules, such as Ca^2+^, Mg^2+^, Zn^2+^, vitamin D (VD), and parathyroid hormone (PTH), are crucial components of bone marrow microenvironment, which may influence the bone marrow osseointegration and the outcome of THA [6]. In addition, human bone marrow cells contain two populations of bone marrow stem cells: mesenchymal stem cells (MSCs also called bone marrow stromal cells) and hematopoietic stem cells (HSCs). Stro-1^+^ is a cell surface antigen expressed by BMSCs [7, 8]. A cell population that is positive for the anti-Stro-1^+^ antibody has been shown to contain BMSC stem cells. The bone marrow Stro-1^+^ is capable of differentiating into multiple mesenchymal lineages including adipocytes, osteoblasts, and chondrocytes [9]. The variation of Stro-1^+^ cells can be observed between human subjects [10]. Thus, we here focused on the association between the Stro-1^+^ cell and the biochemical molecules in the bone marrow microenvironment of THA patients.

Bone morphology proteins (BMPs) are a group of growth factors known for their ability to induce bone formation. To date, over 20 BMP family members have been isolated and characterized [11, 12]. BMPs activate target cells by binding to type-Ia, -Ib, and -II BMP receptors (BMPRs). BMP signals are mediated by type I and type II serine/threonine kinase receptors. These transmembrane receptors recruit and phosphorylate cytoplasmic proteins, especially the receptor-regulated signal transducers Smads 1, 5, and 8 [13]. It has been demonstrated that BMP7 treatment is sufficient to induce all of the genetic markers of osteoblast differentiation in many cell types [14]. The response of human BMSCs to BMP7 is highly diversified, and current clinical studies continue to show a variable success rate of recombinant BMP7 in the treatment of fracture repair and nonunion [15–18]. BMP2 acts as a disulfide-linked homodimer and induces bone and cartilage formation. It is a candidate as a retinoid mediator, and plays a key role in osteoblast differentiation [19]. It is unknown whether the expression of BMP in human BMSCs is correlated with the biochemical molecules in the bone marrow microenvironment of THA patients.

The purpose of this study is to investigate if there is a correlation between the biochemical molecules (Ca^2+^, Mg^2+^, Zn^2+^, VD, and PTH) and the bone marrow osteogenetic factors (Stro-1^+^, BMP2, and BMP7) in the bone marrow microenvironment of THA patients. This study would provide more clues to THA prognosis, and may be useful to determine the surgical strategy, thereby minimizing patient risks.

## Materials and methods

### Study design

A total of 16 consecutive osteoarthritis patients (7 men and 9 women; mean age 59.8 ± 7.2 years, range 52–78 years) and 35 femoral neck fracture patients (18 men and 17 women; mean age 70.8 ± 12.4 years, range 45–89 years) undergoing primary THA in the posterolateral Moore approach (lateral position) performed by the same team of 2 experienced surgeons.

Standardization was conducted as previously described by Lebherz et al. [20], wherein the accuracy of DEXA was confirmed by controlling hip rotation. The current studies were approved by the Institutional Ethics Committee of the Renji Hospital, Shanghai JiaoTong University School of Medicine, and written informed consent was obtained from all patients for their participation.

### Patients

Inclusion criteria were (1) diagnosis of end-stage osteoarthritis or femoral neck fracture; (2) age > 18 years; (3) a surgical candidate for uncemented stems THA (Dorr ≥ 0.75); (4) underwent primary THA with the Smith & Nephew Synergy™ Hip System (Smith & Nephew Advanced Orthopedic Devices, Memphis, TN, USA); and (5) underwent surgery using uncemented stems (Porous Plus HA and a grit blasted) and Reflection™ cup press-fit acetabular components (Smith & Nephew Advanced Orthopedic Devices, Memphis, TN, USA). Exclusion criteria were (1) symptoms or signs of inflammation and infection, rheumatoid arthritis, or another autoimmune disease; or (2) took non-steroidal anti-inflammatory drugs (NSAIDs) within 1 month prior to surgery. Preoperative pain management was done using other types of drugs. This study was approved by the institutional review board of the Renji Hospital (Shanghai, China), and written informed consent was obtained from each participant.

### Surgical sampling

All patients had prosthetic hip replacements. During the surgical procedure, the soft tissues surrounding the femoral head were displaced, after which the femoral head and neck were extracted from the acetabulum manually in all patients to avoid any metal contamination. Then, the sample was cleaned using 1000 mL of physiological saline and stored at − 70 °C for biochemical and radiological examinations.

### Human bone marrow stromal cells

Bone marrow aspirates (5 mL) were obtained from the discarded metaphysis region of the femoral head during THA. The bone marrow aspirates were diluted 1:4 with phosphate-buffered saline (PBS) and layered on Histopaques (Sigma Aldrich, St. Louis, MO, USA) density gradient. Mononuclear cells were isolated by density gradient centrifugation at 600 g for 30 min and washed in PBS. The supernatant (bone marrow aspirate washout) was collected and stored frozen at − 70 °C for the measurement of BMPs. Bone marrow mesenchymal stromal cells (BMSC) collected were then culture-expanded in alpha-modified Eagle’s medium (MEM)/10% fetal bovine serum (FBS) medium. The medium was changed initially at day 4 and then every other day thereafter until the cultures reached confluence. At day 14, the cell was digested with TrypLE Express (Gibco, Grand Island, NY, USA) and collected by centrifugation at 200 g for 10 min. Harvested BMSCs were fixed in 2% (w/v) paraformaldehyde (in PBS) for 15 min, and then used for the following analysis.

### Flow cytometry analysis

Detection of Strol-1^+^, the cell surface marker, was performed by trained technicians blinded to patient identity using Becton Dickinson FACS Calibur Flow Cytometry System (Becton Dickinson, Beckman Coulter, Brea, CA, USA) equipped with Cell Quest software (Beckman Coulter). BMSC cell suspension was incubated with primary antibody for 1 h at 4 °C. Unbound antibodies were removed by washing with PBS. The secondary monoclonal antibodies conjugated with allophycocyanin (APC) were used to detect Stro-1^+^ (BD Pharmingen, San Diego, CA, USA) (1:100 dilution). After incubation, cells were washed and resuspended in 500 L of wash buffer and measured by FACS. The signals corresponding to debris and cell aggregates were first gated out by using the forward light scatter (FSC) and side light scatters (SSC) display. Furthermore, absolute counts of Stro-1^+^ positive cells in BMSC were determined using BD TruCOUNT Tubes (BD Biosciences, San Jose, CA, USA) according to the manufacturer’s instructions. During analysis, the absolute number of Stro-1+ positive cells in cultured BMSC was manually calculated using the following equation: (events of Stro-1^+^ positive cells/events of beads) * (number of beads per test/test volume).

### Enzyme-linked immunosorbent assay

Concentrations of BMP2 and BMP7 in the samples of bone marrow aspirates were determined by commercial enzyme-linked immunosorbent assay (ELISA) kits (R&D System, Minneapolis, MN, USA), following the manufacturer’s instructions. The levels of TRACP5b in the patient’s serum was determined by human tartrate-resistant acid phosphatase 5b (TRACP5b) ELISA kit (Wuhan Saipei Biotechnology Co., LTD., Hubei, China) according to the manufacturer’s instructions. A standard curve was generated and the concentrations (pg/mL; IU/L) of the samples were calculated from the standard curve.

### TRAP staining

Slides were fixed by Fixative solution for 30 s at room temperature, and then thoroughly rinsed in deionized water pre-warmed to 37 °C. The prepared dye solution was added to the dye jar and warmed to 37 °C in a water bath. Slides were added to dye jars and incubated 1 h in 37 °C water bath protected from light. Slides were rinsed thoroughly in deionized water then counterstained 2 min in hematoxylin solution. Slides were rinsed several minutes in alkaline tap water to blue nuclei. After air drying, the slides were sealed with glycerine gelatin and evaluated microscopically.

### Assessment of trace elements

Levels of Ca^2+^, Mg^2+^, and Zn^2+^ in the bone tissue were determined using an atomic absorption spectrophotometer device (Varian AA240FS model; Varian Inc., Belrose, Australia). The measurements were conducted twice for each sample, using light at 2139 nm wavelength according to flame atomization method.

### Measurement of PTH and vitamin D

The measurement of PTH and VD was performed by radio-immune assay (RIA) method with Architect c8000 Clinical Chemistry Analyzer device (Abbott Laboratories. Abbott Park, Illinois, USA).

### DEXA analysis of bone mineral density

**Fig. 1:**
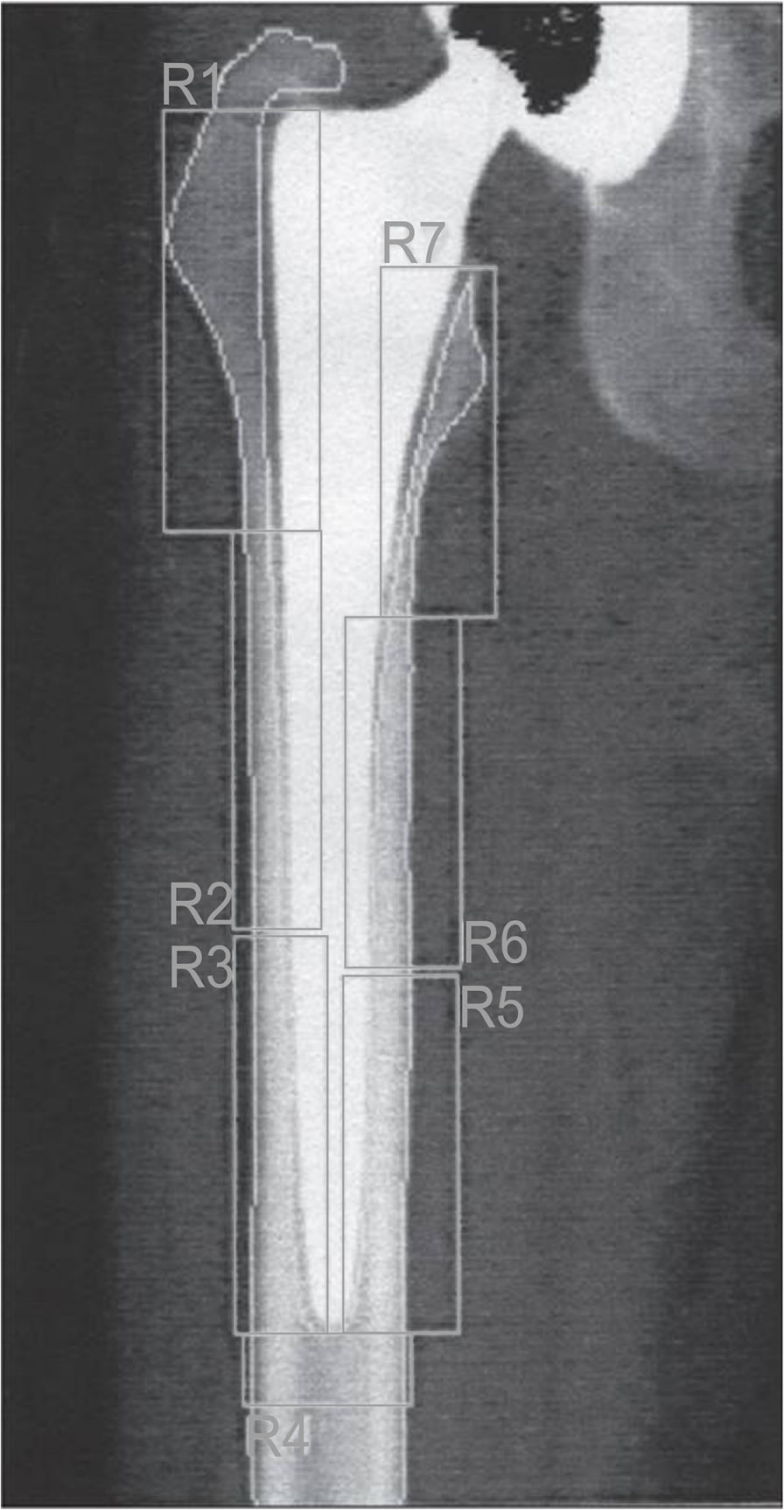
The seven regions of Gruen zones and standardized regions of interest (ROI) used during DEXA analysis

DEXA scans were performed using a HOLOGIC Discovery W (Hologic Inc., Waltham, MA, USA) scanner at 1 week and at 3, 6, and 12 months. Patients were placed in supine position with the affected leg at 10° internal rotation (patella up) with the foot secured in the Hologic foot positioning device to obtain reproducible rotation and thereby limiting measurement errors, as previously described [21]. The femoral stem component and cortical bone were excluded manually during DEXA analysis. Regions of interest (ROI) for each patient were saved using the Hologic image analysis software system (Hologic, Inc., USA) and used for all subsequent measurements. DEXA precision was assessed for all subjects. Bone mineral density (BMD) (g/cm^2^) was determined in the proximal femur regions R1 (greater trochanter region) and R7 zones (calcar region) for each patient using the Gruen zone partition method [22] (Fig. 1).

### Statistical analysis

Patients were stratified as younger (< 70 years) or older (≥ 70 years), or according to gender. All data were analyzed using SPSS 18.0 (SPSS, Inc. Chicago, IL, USA) and expressed as mean ± SD. Variables were compared using one-way analysis of variance (ANOVA) with Newman-Keuls post hoc test (normally distributed) or Mann-Whitney *U* test (non-normally distributed). Correlation analysis was assessed using Spearman’s tests. *P* values < 0.05 were considered to be statistically significant.

## Results

### Patients’ characteristics

All 51 original patients were included in the current study. Clinical and demographic characteristics were reported in Table 1. No patients experienced infection, loosening, or periprosthetic fracture during the 12-month follow-up period. The concentrations of Ca^2+^, Mg^2+^, Zn^2+^, VD, and PTH were not correlated with either age or gender of the THA patients. And according to our previous study, the BMPs and Stro-1^+^ cells were also not associated with the demographic characteristics [23].

**Table 1:** Clinicopathological characteristics of patients

	Features	No. of patients
Gender	Male	25
	Female	26
Age	≥ 70	23
	< 70	28
Diagnosis	Osteoarthritis	16
	Femoral neck fracture	35

### The biochemical molecules and BMP2, BMP7, Stro-1^+^ cells

**Fig. 2:**
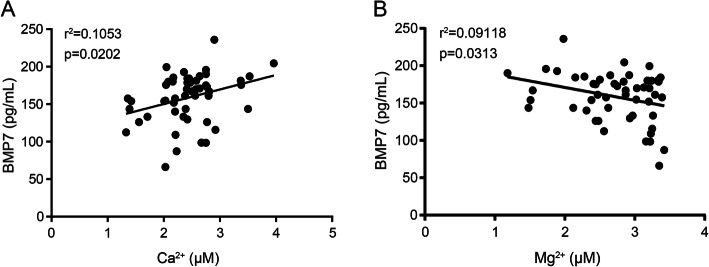
Correlation analysis between the concentrations of Ca^2+^ (**a**), Mg^2+^ (**b**), and the expression of BMP7. The correlation was analyzed by Spearman's test. *P* < 0.05 were considered significant. *R*^2^ values are from correlation coefficient analysis

The samples for biochemical analysis were extracted from the femoral heads collected during surgery. With the data from all measurements, we determined whether each concentration of Ca^2+^, Mg^2+^, Zn^2+^, VD, PTH was correlated with the bone marrow osteogenetic factors (Stro-1^+^ cells, BMP7, and BMP2) (Table 2). By Spearmen’s test, we found that the concentration of Ca^2+^ was positively correlated with the expression of BMP7 (Fig. 2a), but that of Mg^2+^ was negatively correlated with the level of BMP7 (Fig. 2b). The concentrations of Zn^2+^, VD, and PTH were neither correlated with the expressions of BMPs nor the percentage of Stro-1^+^ cells. Therefore, the Ca^2+^-Mg^2+^ axis may be a mediator in the BMP7 related pathways.

**Table 2:** The correlation analysis between the biochemical molecules and the osteogenetic factors

		Ca^2+^ (μM)	Mg^2+^ (μM)	Zn^2+^ (ng/mL)	VD (nM)	PTH (ng/ml)
BMP2	*r* value	− 0.1816	− 0.03489	− 0.04287	0.09361	− 0.09286
	*P* value	0.2022	0.808	0.7652	0.5135	0.5169
BMP7	*r* value	0.32448	− 0.30196	0.16096	0.24256	0.11778
	*P* value	0.0202	0.0313	0.2592	0.0864	0.4104
Stro-1^+^(%)	*r* value	0.28654	− 0.13449	0.19462	0.2683	0.1763
	*P* value	0.0415	0.3467	0.1711	0.05	0.2158

*P* values < 0.05 were considered to be statistically significant

### TRAP content was measured after THA to evaluate osseointegration

**Fig. 3:**
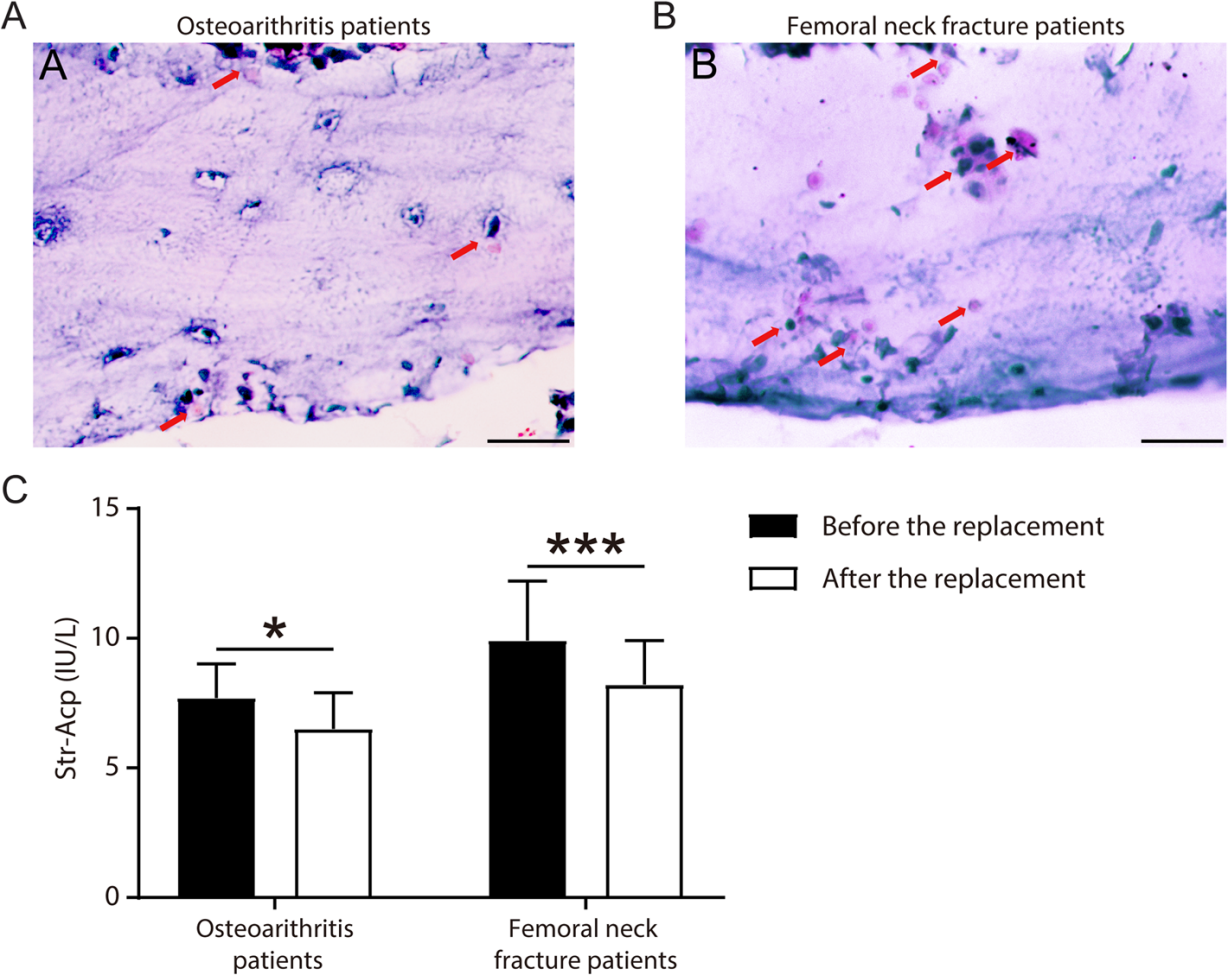
TRAP staining was used to observe the activity of osteoclasts in hip joint tissues of patients with osteoarthritis (**a**) and femoral neck fracture (**b**). Scale bars: μm. **c** Serum TRACP5b levels were detected by ELISA in patients with osteoarthritis and femoral neck fracture before or after THA. *P < 0.05; ***P < 0.001. *P* < 0.05 were considered significant

Osseointegration is not only related to bone formation but also affected by bone resorption. Osteoclasts are closely related to bone resorption capacity. TRAP was a marker for osteoclasts detection. TRAP staining was used to detect the TRAP content in the hip joint specimens from osteoarthritis patients or femoral neck fracture patients (Fig. 3a, b). The results showed that osteoclast activity in the hip joint tissue of patients with femoral neck fracture increased. In addition, compared with the serum TRACP5b detected by ELISA before THA, the results of serum TRACP5b detection in patients after THA were decreased correspondingly (Fig. 3c).

### Ca^2+^, Mg^2+^ and the periprosthetic bone mineral density at 6 months after THA

**Fig. 4:**
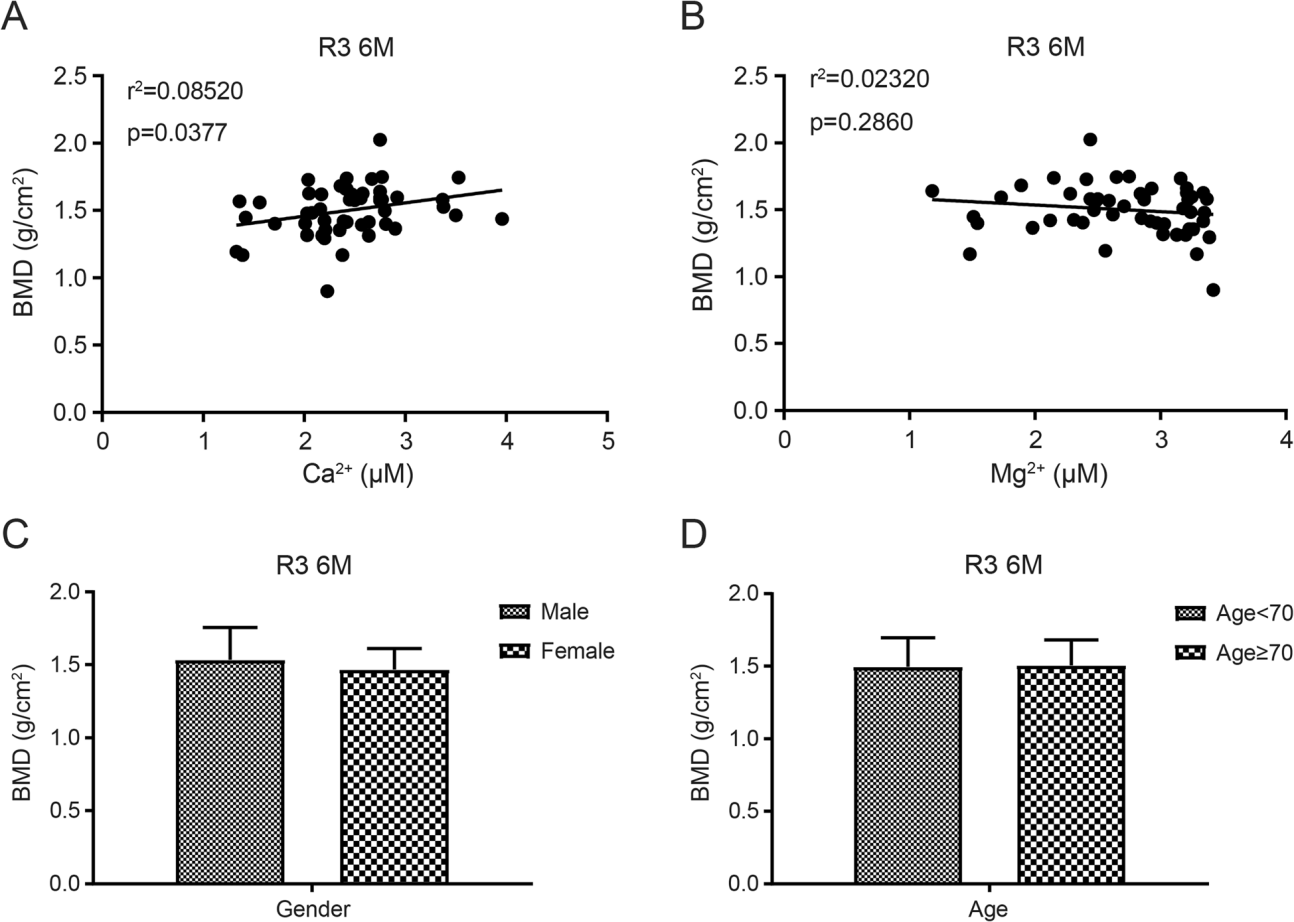
Correlation analysis between the concentrations of Ca^2+^ (**a**), Mg^2+^ (**b**), and the BMD of Gruen R3 at 6 months after THA. The correlation was analyzed by Spearman's test. *P* < 0.05 were considered significant. *R*^2^ values are from correlation coefficient analysis. The BMD was correlated with neither patients’ gender (**c**), nor age (**d**)

Then we wondered if the concentrations of Ca^2+^ and Mg^2+^ are correlated with the postoperative bone mineral density (BMD) of THA patients. According to Gruen zone partition method, we found the BMD of R3 at 6 months after THA was positively correlated with the concentration of Ca^2+^, but not with that of Mg^2+^ (Fig. 4a, b). These results indicate that the concentration of Ca^2+^ may be an indicator for the prognosis of THA patients. Meanwhile, gender or age was irrelevant with R3 BMD by the statistics analysis (Fig. 4c, d).

### Multiple linear regression analysis of the postoperative osteointegration

Due to no obvious bone resorption from the frontal and lateral X-ray films, we consider the periprosthetic BMD as indicator of postoperative osteointegration. Correlation analysis was performed by method of multivariate linear regression and stepwise variables selection, the postoperative bone mineral density as the dependent variable and the independent variable by stress shielding and Ca^2+^ concentration. Results showed that the independent variable Ca^2+^ concentration was positively correlated with postoperative bone integration (Table 3).

**Table 3:** Multiple linear regression analysis of the postoperative osteointegration

Variables	Estimated regression coefficient	Standard deviation	*t*	*P*	Regression coefficient
Intercept	3.98612	10.26322	0.42	0.6653	0
stress shielding	0.76531	0.30296	2.98	0.0241	0.32176
Ca_2_+ concentration	0.48289	0.28563	3.82	0.0102	0.42299

*P* values < 0.05 were considered to be statistically significant

## Discussion

THA has proved to be an excellent and reliable treatment procedure for the end stages of hip pathology since the 1960s. However, the periprosthetic loss of BMD and subsequent loss of bone tissue in the proximal femur are common in the first year following THA [24]. The lost bone tissue is usually not recovered [25]. Furthermore, the severe periprosthetic bone loss may contribute to complications such as aseptic loosening of the prosthesis and an increased risk of periprosthetic fracture [26]. Therefore, implant failure and periprosthetic fractures because of periprosthetic bone loss are a major concern in THA [27]. Successful osseointegration relies on the interplay of implant surface and periprosthetic bone marrow composition. A favorable bone marrow environment should have sufficient osteoprogenitor cells able to differentiate into osteoblasts in response to systemic or local growth factors. Meanwhile, trace elements, VD, and PTH are also key components in the bone marrow microenvironment, which involve the bone remodeling process with different roles. A better understanding of the association of these biochemical molecules with the bone marrow composition is critical in predicting the outcomes of post-THA implant fixation and implant longevity.

Few studies have been performed to understand the impact of the biochemical molecules on the bone marrow contents of BMPs and Stro-1^+^ cells, and the potential influence on the postoperative BMD of THA patients. Our data showed that the concentration of Ca^2+^ was positively correlated with BMP7 expression, and with the postoperative BMD of Gruen zone R3. However, the concentration of Mg^2+^ had little impact on the R3 BMD, although it was negatively correlated with the expression of BMP7. Our data also suggested that the other biochemical molecules, such as Zn^2+^, VD, and PTH, were not significantly correlated with any bone marrow osteogenetic factors (BMP2, BMP7, Stro-1^+^ cells).

BMPs are a group of growth factors known for their ability to induce bone formation, and over 20 BMP family members have been isolated and characterized so far [28]. BMPs interact with specific receptors on the cell surface, referred to as BMP receptors (BMPRs) [29]. BMP signals are mediated by type I and type II serine/threonine kinase receptors. Two type I receptors have been identified: BMPR1a (ALK3) and BPMR1b (ALK6). BMPR1a is necessary for the extracellular matrix deposition by osteoblasts [30]. The type II receptor (BMPR2) binds BMPs, and the signaling begins with the binding of a BMP to the BMPR2. This causes the recruitment of a BMP type I receptor, which it phosphorylates. BMP7 plays a key role in the transformation of BMSC into bone and cartilage [31]. It has been demonstrated that BMP7 treatment is sufficient to induce all of the genetic markers of osteoblast differentiation in many cell types. BMP7 has been used in clinical applications to accelerate fracture healing, to treat established nonunions, to enhance primary spine fusions, and to treat large bone-loss defects [32]. The responses of human BMSC to BMP2 are highly diversified and current clinical studies continue to show a variable success rate of recombinant BMP7 in the treatment of fracture repair and nonunion [33]. BMPs play a critical role in controlling implant osseointegration [34]. Osseointegration is not only related to bone formation but also affected by bone resorption. Osteoclasts are closely related to bone resorption, and TRAP is an indicator to detect osteoclasts. Therefore, we can evaluate the osseointegration after THA by detecting the content of TRAP. TRAP staining showed that osteoclast activity increased in the hip joint tissue of patients with femoral neck fractures. The ELISA results showed that the serum TRACP5b levels in patients with osteoarthritis or femoral neck fractures decreased after THA, which demonstrated that the osteoclast activity in patients after THA was decreased.

In the skeleton, magnesium supports the production of hydroxyapatite [35], bone marrow stromal cells mineralization [36], and active VD synthesis. Thus, magnesium deficiency via hypocalcemia elevates parathormone synthesis and subsequently osteoclast activity. Zinc is a growth stimulator through activation of enzymes, which support synthesis of DNA, RNA, and proteins. Zinc increases osteoblastic activity and promotes the synthesis of collagen [37]. In addition, zinc inhibits osteoclastic bone resorption and thus disconnects bone remodeling in favor of bone formation [38]. It is known that a normal calcium balance together with a normal VD status is important for maintaining well-balanced bone metabolism, and for many years, calcium and VD have been considered crucial in the prevention and treatment of osteoporosis [39]. In bone, PTH enhances bone resorption through stimulating the OPG/RANKL/RANK pathway [40]. Our statistical results showed that calcium concentration was positively correlated with osseointegration after THA. However, there are still several limitations to the present study. Such as, an extended follow-up would make more sense in confirming these results. In addition, the samples in the present study were extracted from the femoral head removed during the surgery, which cannot eliminate traumatic influences brought by the surgery.

## Conclusions

Taken together, a better understanding of the correlations about bone marrow composition in THA patients will help clinicians to predict and prevent post-THA implant failure due to the disruption of osseointegration.

## Data Availability

Not applicable.
